# Efficacy of cartilage-targeted IGF-1 in a mouse model of growth hormone insensitivity

**DOI:** 10.3389/fendo.2024.1523931

**Published:** 2025-01-09

**Authors:** Krishma Tailor, Janine van Ree, Timothy Stowe, Brit Ventura, Connor Sisk, Joanna Courtis, Anna Camp, Fatima Elzamzami, Jan van Deursen, Robert O’Brien, Jeffrey Baron, Julian C. Lui

**Affiliations:** ^1^ Section on Growth and Development, Eunice Kennedy Shriver National Institute of Child Health and Human Development, National Institute of Health, Bethesda, MD, United States; ^2^ Cavalry Biosciences, Inc., San Francisco, CA, United States

**Keywords:** short stature, matrilin-3, GH-insensitivity syndrome, hypoglycemia, drug targeting

## Abstract

Recombinant human IGF-1 is used to treat severe primary IGF-1 deficiency, but this treatment requires twice-daily injection, often does not fully correct the growth deficit, and has important off-target effects. We therefore sought to target IGF-1 to growth plate cartilage by generating fusion proteins combining IGF-1 with single-chain human antibody fragments that target matrilin-3, a cartilage matrix protein. We previously showed that this cartilage-targeting IGF-1 fusion protein (CV1574-1) promoted growth plate function in a GH-deficient (lit) mouse model. Here, we studied CV1574-1 in a second mouse model, C57BL/6 wild-type mice treated with pegvisomant to induce GH resistance. In this model, once-daily injections of CV1574-1 for 5 days partially restored the pegvisomant-induced decrease in growth plate height without increasing kidney cell proliferation. Furthermore, we found that subcutaneous CV1574-1 showed significantly reduced hypoglycemic effect compared to injection of IGF-1 itself. Lastly, to gain mechanistic insights into the role of matrilin-3 targeting, we assessed the ability of CV1574-1 to activate AKT signaling *in vitro* and found that CV1574-1 caused a prolonged increase in AKT signaling compared to IGF-1 and that this effect was dependent on matrilin-3. Taken together, our findings provide further evidence that cartilage-targeted therapy could provide new pharmacological approaches for the treatment of childhood growth disorders, such as GH-insensitivity syndrome.

## 1 Introduction

Longitudinal bone growth in children is driven by a cartilaginous structure near the ends of long bones termed the growth plate (1). In the growth plate, chondrocyte proliferation followed by hypertrophic differentiation leads to chondrogenesis. The newly formed cartilage matrix provides a scaffold on which incoming osteoblasts form new bone. The net result is bone elongation and consequently height gain (2).

Growth plate chondrogenesis is regulated by multiple factors, including intracellular, extracellular matrix-related, paracrine, and endocrine regulatory systems. Consequently, in children, disturbances in these regulator systems that decrease growth plate chondrogenesis result in short stature (3). Examples of endocrine regulators that are important for childhood bone growth include growth hormone (GH) and insulin-like growth factor-I (IGF-1), both of which promote chondrocyte proliferation and differentiation in the growth plate (4). GH is produced by the pituitary gland in the brain and acts on the liver to stimulate hepatic production of IGF-1. Both circulating GH and IGF-1 then act on the growth plate as endocrine growth factors. In addition, GH also stimulates the local production of IGF-1 at the growth plate, which acts as a paracrine growth factor (5). Consequently, short stature occurs in patients with either GH deficiency or with GH insensitivity due to genetic variants in the GH receptor.

Currently, the most commonly used treatment for children with short stature is recombinant human GH therapy. The FDA approved the use of GH in treating a number of causes of short stature, including growth hormone deficiency, Turner syndrome, Prader Willi syndrome, Noonan syndrome, chronic renal disease, children born small for gestational age with inadequate catch-up growth, and idiopathic short stature (6). The potential adverse effects of GH treatment include increased intracranial pressure, slipped capital femoral epiphysis, insulin resistance, and a theoretical increased risk of malignancies (7, 8).

In 2005, the FDA approved IGF-1 therapy (administered as a twice-daily subcutaneous injection) for treatment of patients with severe primary IGF-1 deficiency (IGFD) (in whom GH therapy is not effective), many of whom have GH resistance due to variants in the GH receptor (Laron syndrome or GH insensitivity syndrome), defects in the post-GH receptor signaling pathway, or variants in the IGF-1 gene (9, 10). However, IGF-1 treatment has significant potential adverse effects including hypoglycemia, lymphoid overgrowth, benign intracranial pressure, coarsening of facial features, and, similar to GH, a theoretical increased risk of malignancies (11–13).

Because both GH and IGF-1 have limited efficacy at the growth plate in many conditions and adverse effects on other tissues, we previously developed single-chain variable antibody fragments (scFvs) targeting cartilage matrix protein matrilin-3 (14) for delivery of chondrogenic growth factors locally at the growth plate. We have shown that fusion proteins combining IGF-1 with these scFvs augmented the therapeutic efficacy of IGF-1 at the growth plate and reduced off-target effects on kidney (a non-targeted tissue) in a GH-deficient lit mouse model (15).

In the current study, we tested the efficacy of these IGF-1 fusion proteins using a second *in vivo* model, in which we induced GH deficiency in mice by injection of pegvisomant. Pegvisomant (B2036-PEG) is a GH receptor antagonist which decreases the production of IGF-1 (16, 17). It is an FDA-approved drug for treatment of GH excess in acromegaly patients (18). We hypothesized that pegvisomant-treated wild-type mice could serve as an *in vivo* model for GH-insensitivity syndrome to assess the efficacy of cartilage-targeted IGF-1 fusion protein (hereafter termed CV1574-1). In addition, we assessed the hypoglycemic effect of CV1574-1 and investigated the mechanisms by which targeting matrilin-3 could prolong or improve IGF-1 receptor activation.

## 2 Materials and methods

### 2.1 Animals

C57BL/6 male and female wild-type mice were obtained from Charles River Laboratory. The National Institute of Child Health and Development Animal Care and Use Committee approved all animal procedures.

### 2.2 Generation of cartilage-targeted IGF-1 fusion protein CV1574-1

CV1574-1 is a fusion protein combining IGF-1, a monomeric antibody Fc region, and an ScFv targeting matrilin-3. Expression and purification were performed at Fusion Antibodies in Belfast, Northern Ireland. DNA coding for 1574-1 was cloned into the proprietary vector pETE V2. Secreted protein was generated using transient expression in an HEK-based system. At harvest, media was clarified and filtered followed by affinity purification by AKTA FPLC (Amersham Pharmacia Biotech). Protein was polished by size exclusion chromatography using a Superdex 200 Increase 10/300 GL column (GE Healthcare) to remove high molecular weight aggregates and ensure monodispersity of >90%.

### 2.3 Pharmacokinetic characterization of cartilage-targeted IGF-1 fusion protein CV1574-1

A single subcutaneous injection of CV1574-1 molecule at a 12 mg/kg dose was performed in 1-week-old C57BL/6 pups (n=3 pups/group). Proximal tibial epiphyseal cartilage tissue and the heart were harvested at 4 hours and 24-hour time points, and the accumulation of CV1574-1 in tissue (**Supplementary Figures 1A, B**) was measured using a hIgG ELISA (Abcam, Cat# ab195215). Adult C57BL/6 WT mice received a subcutaneous injection of 1.5 mg/kg of CV1574-1 (n=3 mice/group). Blood was collected at 1 hour, 4 hours, 24 hours, and 72 hours. CV1574-1 in plasma was measured by hIgG ELISA assay (Abcam, Cat# ab195215) (**Supplementary Figure 1C**).

### 2.4 Development of GH-resistant mouse model by pegvisomant administration

**Figure 1:**
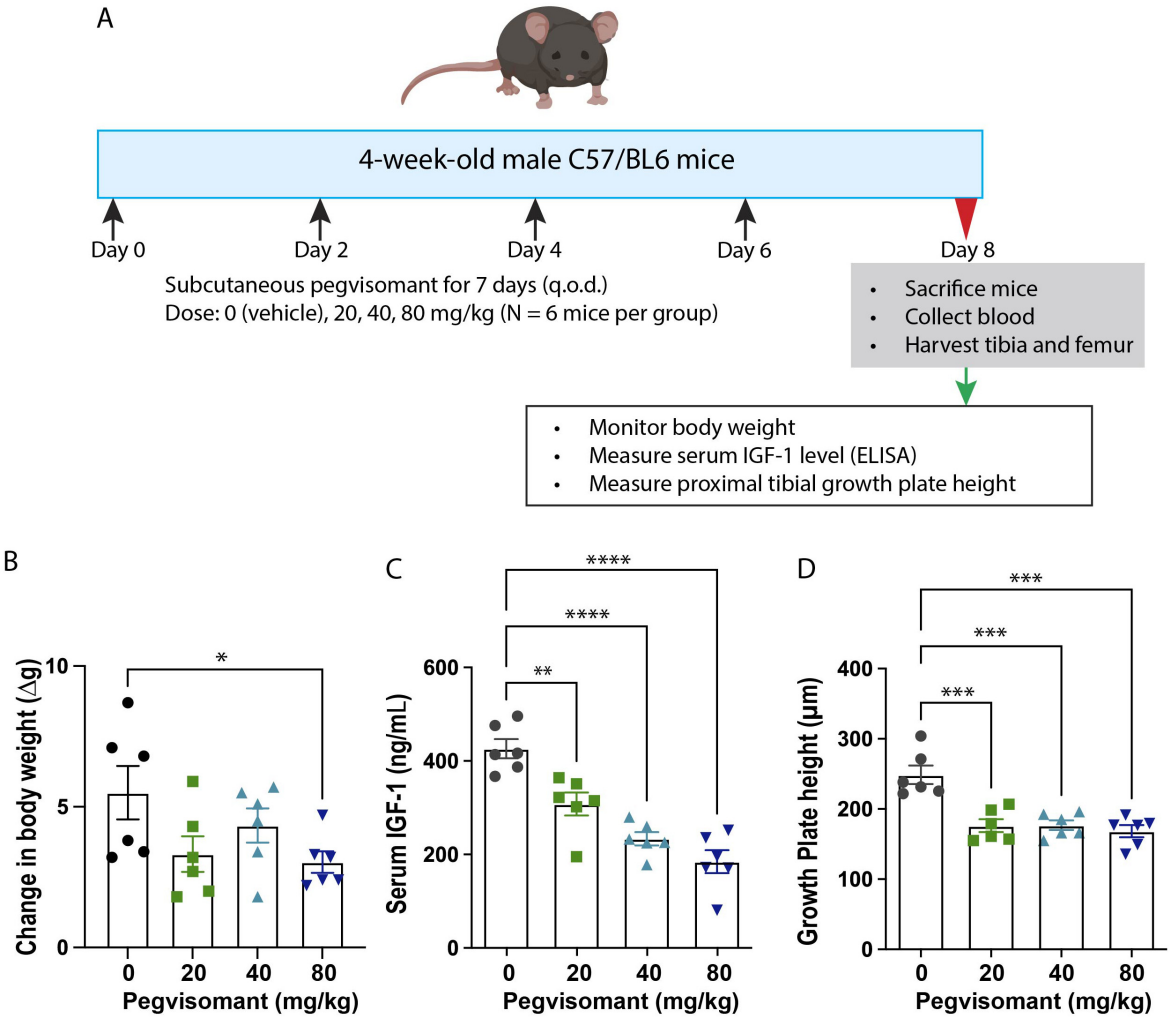
Pegvisomant treatment suppressed body growth, serum IGF-1 and growth plate activity in wildtype male mice. **(A)** Experimental design of pegvisomant dose-finding study in C57BL/6 male mice. Treatment with pegvisomant decreased body weight gain **(B)**, serum IGF-1 **(C)**, and overall growth plate height of proximal tibia **(D)**. Bar graphs represent mean± SEM, *P<0.05, **P<0.01, ***P<0.001, **** P<0.0001 by one-way ANOVA, followed by pairwise comparison with the saline group. P-value corrected for multiple comparisons by Dunnett’s test. N=6 mice per group.

To create a GH-resistant model, three different concentrations of pegvisomant (FDA-approved formulation; Pfizer; 20 mg/mL; doses, 0 (vehicle), 20 mg/kg, 40 mg/kg, or 80 mg/kg of body weight) were injected subcutaneously in C57BL/6 male mice (4 weeks of age, 6 mice per group) every other day for 7 days (**Figure 1A**). We assessed body weight before each injection. On day 8, mice were sacrificed, and serum IGF-1 and proximal tibia growth plate height were measured (see methods below). A dose that adequately decreased serum IGF-1 level and growth plate height was chosen to test the efficacy of IGF-1 and CV1574-1.

### 2.5 Efficacy of CV1574-1 (IGF-1 fusion protein) in the GH-resistant mouse model

**Figure 2:**
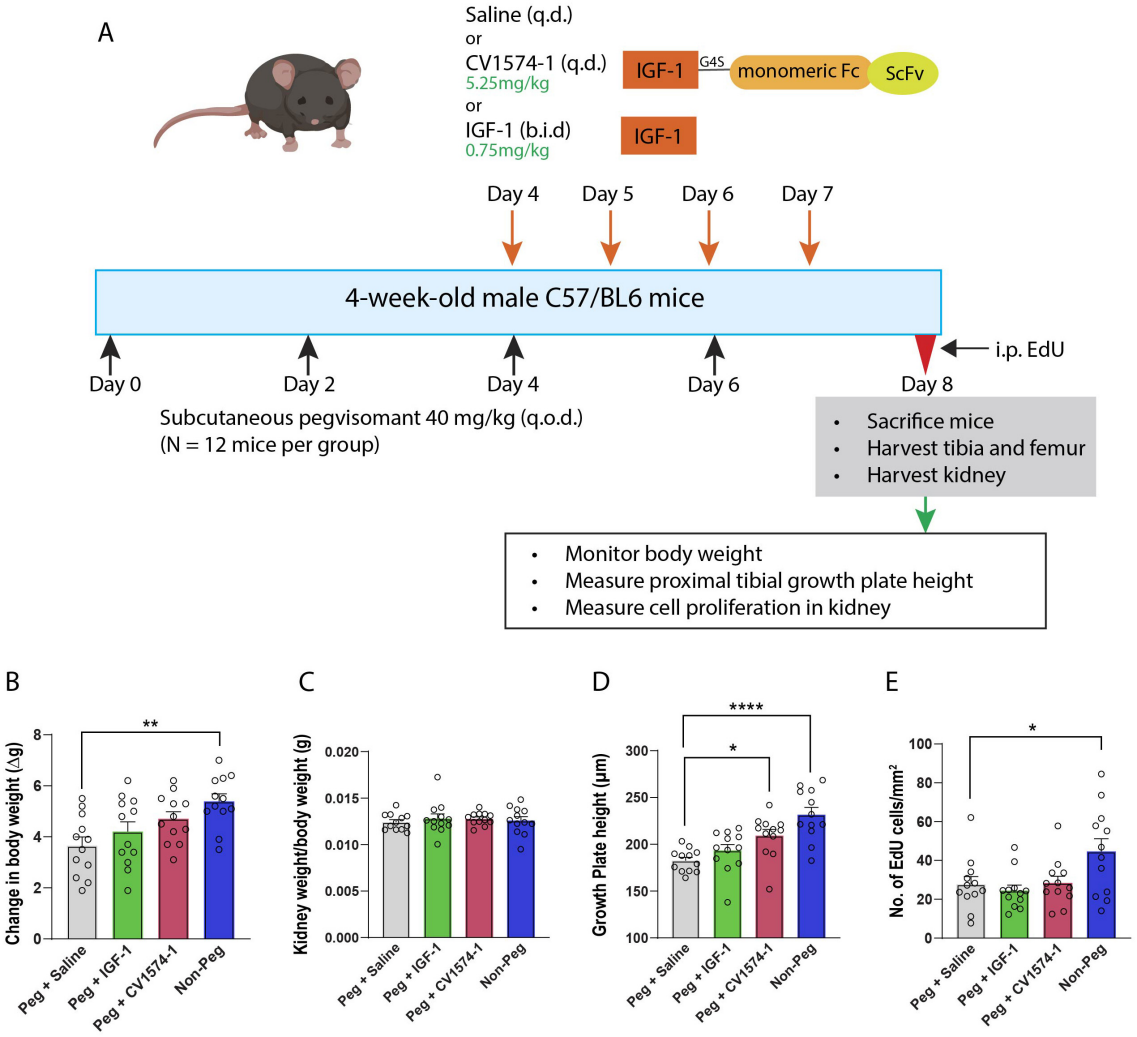
Effects of CV1574-1 in the pegvisomant-induced GH-resistant mouse model. **(A)** Experimental design. Body weight **(B)**, kidney weight/body weight **(C)**, overall growth plate height of proximal tibia **(D)**, and cell proliferation assay in kidney **(E)** in male mice treated with either 40 mg/kg pegvisomant (Peg) plus either saline, twice-daily IGF-1, or once daily CV1574-1, and in male mice not treated with pegvisomant (Non-Peg). Bar graphs represent mean± SEM, *P<0.05, **P<0.01, ****P<0.0001 by one-way ANOVA followed by multiple comparisons between all groups. P-value corrected by Dunnett’s test. N=12 mice per group.

Pegvisomant (40 mg/kg) was administered subcutaneously in 4-week-old male mice (N=12 per group) on alternate days. Starting on day 4, saline (negative control), 0.75 mg/kg (=98nmol/kg) IGF-1 twice a day (positive control), or 5.25 mg/kg (=98nmol/kg) CV1574-1 given once a day were administered subcutaneously for 5 consecutive days while continuing the alternate day pegvisomant treatment (**Figure 2A**). One group of naïve mice without pegvisomant or IGF-1 treatment was included for comparison. Body weight was measured daily during treatment. At the end of the treatment, mice were sacrificed, and the kidneys and tibias were harvested. The proximal growth plate height was measured, and cell proliferation in the kidney was assessed using 5-ethynyl-2’-deoxyuridine (EdU, see details below).

### 2.6 Measurement of serum IGF-1 level

The levels of mouse serum IGF-1 were measured by a quantikine ELISA kit (R&D Systems, Cat# MG-100).

### 2.7 Measurement of proximal tibia growth plate height

After the mice were sacrificed, tibias were fixed in 10% buffered formalin (Electron Microscopy Sciences), and tibias were decalcified in 0.5 M EDTA (pH 7.4) for at least 2 weeks before sectioning. Tibias were then paraffin-embedded, and 10 µm sections were mounted on Superfrost Plus slides (Fisher Scientific). Histological evaluations were performed on Masson Trichrome-stained sections and visualized using a slide scanner (Nanozoomer S60 digital slide scanner, Hamamatsu) under bright-field microscopy. All histological measurements were performed in the central two-thirds of the proximal tibial growth plate using Nanozoomer software NDP.view2 (RRID : SCR_025177). For each growth plate section, we performed approximately 10 measurements of overall growth plate height, and 24 sections were analyzed per animal.

### 2.8 EdU staining for measurement of cell proliferation in kidney

To assess cell proliferation, EdU (Sigma-Aldrich; 0.1 mg/g body mass), was injected intraperitoneally 2 hours before mice were sacrificed. Whole kidneys were dissected, formalin-fixed, and embedded in OCT (optimal cutting temperature) compound (Tissue-Tek) and frozen on dry ice. Sagittal sections were made close to the middle of the kidney and mounted on Superfrost Plus slides. EdU labeling was detected using the Click-it Alexa fluor 647 EdU imaging kit (Invitrogen). Sections were visualized using a Keyence 700 by fluorescence microscopy. EdU-positive cells were counted using hybrid cell count software. For each animal, 6 kidney sections were analyzed.

### 2.9 Measurement of serum glucose level

C57BL/6 female WT mice (8-10 weeks old) were fasted for 2 hours prior to the start of the experiment and throughout the duration of the experiment with access to water only. Mice received single subcutaneous injection of saline, 0.3mg/kg (=39nmol/kg) of IGF-1, or 2.7mg/kg (=39nmol/kg) of CV1574-1, followed by blood sampling predose (0 min), 20 min, 40 min, 1 hour, 2 hours, and 3 hours post-injection. Blood glucose levels were measured using an Alphatrak3 blood glucose meter (Zoetis).

### 2.10 Construction of stable matrilin-3 knockout cell line (CRISPR-CAS9)

MATN3 was ablated in rat chondrosarcoma cells using the Alt-R CRISPR-Cas9 System (IDT) according to the manufacturer’s protocol with Matn3-targeting gRNA oligo/AltR1/rCrA rUrUrG rArCrA rCrUrC rUrGrG rArCrA rUrCrG rGrUrU rUrUrA rGrArG rCrUrA rUrGrC rU/AltR2/and Lipofectamine CRISPRMAX transfection reagent (Thermo Fisher Scientific CMAX00008). In the resulting RCS-MATN3 KO cells the absence of MATN3 was verified by immunofluorescence staining with anti-MATN3 (**Supplementary Figure 3A**) and Western blot analysis with anti-mMATN3 antibody (R and D Systems Cat# AF3357, RRID : AB_2141517) (**Supplementary Figure 3B**).

### 2.11 pAKT measurement in rat chondrosarcoma cells

Approximately 75,000 wild-type or matrilin-3 knock-out RCS cells were plated per well of a 24-well cell culture plate. Cells were serum starved (0.2% BSA in DMEM/F12) for ~6.5 hours, before treating with 12.5 nM IGF-1 or 12.5 nM CV1574-1 for 20 min. Cells were then washed with PBS to remove unbound IGF-1 and CV1574-1 fusion protein, followed by incubation in starvation medium (0.2% BSA in DMEM/F12). Cells were then collected at time 0 min, 15 min, 30 min, 1 hour, 2 hours, and 4 hours for measurement of pAKT by ELISA (R&D Systems, DYC887B-5).

### 2.12 Statistical analysis

Data are presented as mean ± SEM. Line graphs and bar graphs were generated with GraphPad Prism. One-way ANOVA analyses, two-way ANOVA, and Tukey’s multiple comparisons test were performed using GraphPad Prism.

## 3 Results

### 3.1 Pegvisomant treatment suppressed body growth, serum IGF-1 and growth plate activity in wild-type male mice

To develop a GH-resistant mouse model, 4-week-old C57BL/6 wild-type male mice were treated for 8 days with saline or pegvisomant 20 mg/kg, 40 mg/kg, and 80 mg/kg on alternate days (**Figure 1A**). Pegvisomant treatment tended to slow the increase in body weight compared to saline, with 80 mg/kg reaching statistical significance (**Figure 1B**). Serum IGF-1 level was measured as an indicator of GH action at the liver (19–22). Treatment with pegvisomant significantly reduced serum IGF-1 level in a dose-dependent manner (**Figure 1C**); pegvisomant doses of 20 mg/kg, 40 mg/kg, and 80 mg/kg significantly reduced IGF-1 levels to 72%, 55%, and 43% respectively, compared to the saline control. Our previous study established that overall growth plate height is a reliable readout for IGF-1 at the growth plate (15). Therefore, we used overall growth plate height as another endpoint to assess the effect of pegvisomant. All three doses of pegvisomant significantly decreased total growth plate height by ~30% compared to saline (**Figure 1D**). Based on these observations, we selected a 40 mg/kg pegvisomant dose for subsequent experiments because it showed significant reduction in serum IGF-1 and growth plate height.

### 3.2 CV1574-1 showed efficacy at the growth plate without increasing kidney proliferation in a GH-resistant mouse model

Next, we examined the dose-response effect of cartilage-targeted IGF-1 fusion protein CV1574-1 in the GH-resistant model. Pegvisomant-treated mice were injected with three different doses (0.58 mg/kg, 1.75 mg/kg or 5.25 mg/kg) of CV1574-1(**Supplementary Figure 2A**). We found that CV1574-1 increased gain in body weight and growth plate height (**Supplementary Figures 2B, C**). We next compared the effect of saline, once daily injection of CV1574-1 (5.25mg/kg), or twice daily injection of IGF-1 (0.75mg/kg, molar equivalent to 5.25mg/kg CV1574-1) in pegvisomant-treated mice. An additional control group of mice not treated with pegvisomant or IGF-1 was also included for estimating the effect of CV1574-1 or IGF-1 on ameliorating pegvisomant-induced growth suppression. First, we found that mice not treated with pegvisomant have significantly more body weight gain compared with mice treated with pegvisomant and saline (**Figure 2B**). When pegvisomant-treated mice were treated with IGF-1 or CV1574-1, the gain in body weight appeared to be improved compared with saline, although not reaching statistical significance (**Figure 2B**). We observed no significant difference in kidney weight divided by body weight between the mice in the control group and CV1574-1 or IGF-1 treatment groups (**Figure 2C**). Interestingly, the once-daily injection of CV1574-1 significantly increased the overall growth plate height compared to saline (**Figure 2D**), while the IGF-1 twice-daily injection showed a tendency towards increased growth plate height compared to saline but did not reach statistical significance.

Next, we assessed the off-target effect of CV1574-1 on tissues other than the growth plate by performing a cell proliferation assay in the kidney. Mice treated with pegvisomant (and saline) showed less EdU-labeling in kidney cells compared to mice not receiving pegvisomant. (**Figure 2E**). In the pegvisomant-treated mice, neither the once-daily injection of 5.25 mg/kg CV1574-1 nor twice daily IGF-1 showed an increase in EdU labeling compared to the saline-treated group. Collectively, these results are consistent with our previous study in the lit mouse model and further support the hypothesis that cartilage-targeted IGF-1, such as fusion protein CV1574-1, promote growth locally at the growth plate with limited off-target effects on other tissues. In extension of our previous findings, the current study indicates that cartilage-targeted IGF-1 was effective not only in a GH-deficient model but also in a GH-resistant model.

### 3.3 Subcutaneous CV1574-1 showed reduced hypoglycemic effect compared to IGF-1

**Figure 3:**
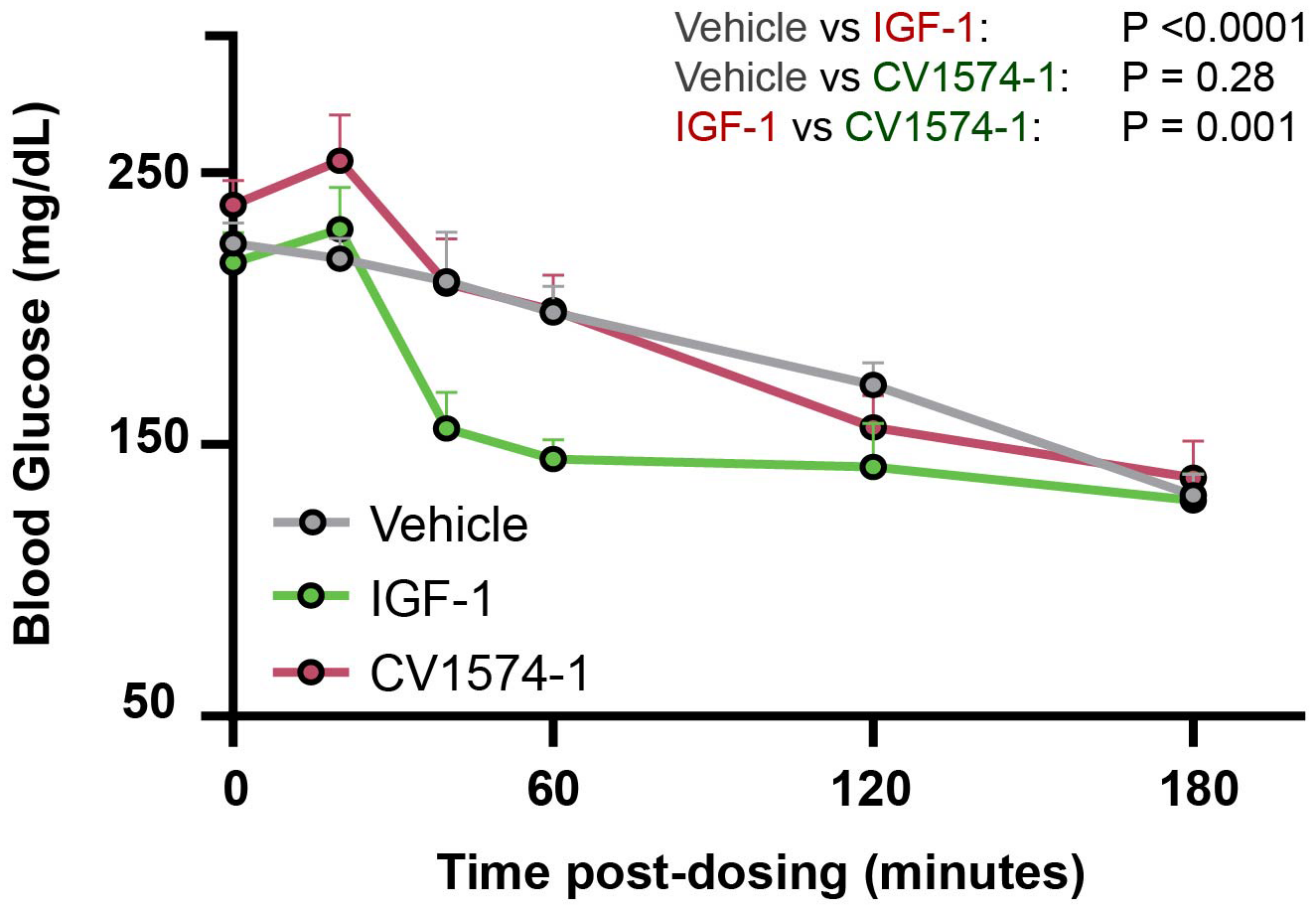
CV1574-1 showed reduced hypoglycemic effect compared to IGF-1. 8-10-week-old C57BL/6 female WT mice were fasted for 2 hours and then received a single subcutaneous injection of vehicle, 39 nmol/kg CV1574-1 or 39 nmol/kg IGF-1. Serum glucose was measured at the time points indicated. Mean ± SEM. Statistical analysis was performed by repeated measures ANOVA time and treatment as sources of variation. N=4 mice per group.

One adverse effect of IGF-1 treatment is hypoglycemia due to its insulin-like action (23). Therefore, we compared the effects of IGF-1 and CV1574-1 on blood glucose levels in fasting mice. A single subcutaneous injection of IGF-1 (0.3mg/kg) decreased blood sugar compared to saline or CV1574-1 (2.7mg/kg) (**Figure 3**). Notably, subcutaneous CV1574-1 did not lower the blood glucose levels compared to saline (**Figure 3**), indicating that subcutaneous CV1574-1 has less tendency to induce hypoglycemia than does IGF-1.

### 3.4 CV1574-1 showed prolonged IGF-1 receptor activation *in vitro*

Finally, we aimed to investigate the molecular mechanisms by which cartilage-targeting could enhance IGF-1 growth-promoting activity locally. We hypothesized that targeting IGF-1 to matrilin-3 leads to increased retention time of IGF-1 in the growth plate, allowing prolonged IGF-1 receptor activation and increased *in vivo* efficacy. Previous studies have demonstrated that IGF-1 receptor activation positively regulates chondrocyte differentiation and proliferation and that AKT phosphorylation is involved in one of the IGF-1 signal transduction pathways. Therefore, we investigated the ability of CV1574-1 to induce AKT phosphorylation in rat chondrosarcoma (RCS) cells.

**Figure 4:**
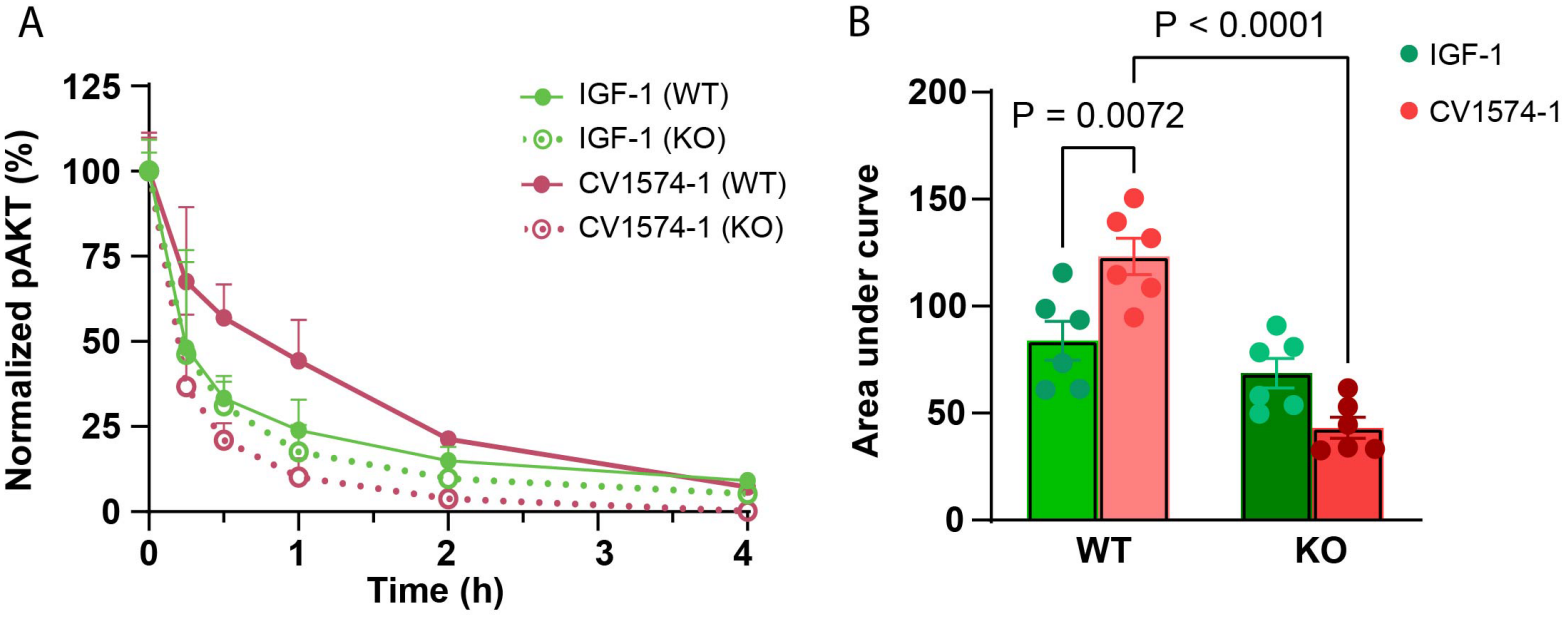
CV1574-1 showed prolonged IGF-1 receptor activation *in vitro*. **(A)** Serum-starved wild-type rat chondrosarcoma (RCS) and matrilin-3 knock-out (KO) RCS cells were treated transiently with 12.5 nM IGF-1 or CV1574-1 for 20 minutes, followed by washout with PBS to remove unbound IGF-1 and fusion protein. The lysates were prepared at indicated time points. pAKT signal was measured in cell lysates by ELISA. Mean ± SEM (N=6). **(B)** The bar graph represents the area under the curve (AUC) value for pAKT signal from **(A)**. P-values were generated by one-way ANOVA comparing the effect of treatment (IGF-1 versus CV1574-1) within the same cell type (WT or matrilin-3 KO RCS cells) and comparing the effect of cell types (WT versus KO) for the same treatment (by IGF-1 or CV1574-1). AUC values were generated using GraphPad Prism.

RCS cells were transiently exposed to 12.5 nM CV1574-1 or IGF-1, followed by a washout period. We found that CV1574-1 induced a significantly prolonged pAKT signal compared to IGF-1 (**Figure 4A**, solid lines, red versus green; **Figure 4B**, red versus green, WT). Importantly, when the same experiment was performed using matrilin-3 knockout RCS cells, CV1574-1 no longer showed an increase in pAKT signaling compared with IGF-1 (**Figure 4A**, dotted lines, red versus green; **Figure 4B**, red versus green, KO). Additionally, CV1574-1-induced (but not IGF-induced) pAKT signaling was significantly decreased in matrilin-3 KO cells compared to WT cells (**Figure 4B**, red bars, WT versus KO), suggesting CV1574-1-induced pAKT signal is dependent on matrilin-3 binding. These findings are consistent with the hypothesis that matrilin-3 binding resulted in longer retention time in proximity to the cells, allowing prolonged IGF receptor signaling *in vitro*.

## 4 Discussion

In the current study, we developed a growth hormone-resistant mouse model using a GH receptor antagonist, pegvisomant, and then used that model to test the efficacy of IGF-1 fusion protein CV1574-1. Various studies have reported different pegvisomant doses in mice to achieve biological activity (20–22). Here, we showed that pegvisomant reduced serum IGF-1 level decreased growth plate height, and decreased body weight gain in C57BL/6 wild-type male mice, suggesting pharmacological inhibition of GH action. We then asked whether twice-daily IGF-1 or once-daily cartilage-targeted IGF-1 fusion protein CV1574-1 could rescue the growth inhibition of pegvisomant (40 mg/kg) treatment. Twice-daily injection of IGF-1 was used because our previous study in lit mice had shown that once-daily injection had limited, if any, efficacy on the growth plate, while similarly, once-daily injection of CV1574-1 was used because our previous work in lit mice indicated that to be efficacious. Here, we found that a once-daily injection of CV1574-1 (5.25 mg/kg) partially rescued the suppression in growth plate height and body weight gain in pegvisomant-treated mice, similar to our previous findings in the lit mouse model (15). Importantly, this growth-promoting effect of CV1574-1, administered only once daily, appeared to be at least as prominent as twice-daily IGF-1 injection. The findings are consistent with the hypothesis that cartilage-targeting improves the efficacy of IGF-1 at the growth plate, although other differences between the fusion protein and IGF-1 alone could be responsible for this augmented growth-promoting effect. When we assessed the off-target effects of IGF-1 treatment, we found that pegvisomant-treated mice showed significantly lower EdU labeling in the kidney compared to naive mice, indicating that pegvisomant decreased kidney cell proliferation. Once-daily treatment of CV1574-1 fusion protein did not increase cell proliferation in the kidney compared to saline, which is consistent with our previous findings in the lit mice. However, in the current study, we also did not observe any increase in kidney cell proliferation after twice-daily IGF-1 treatment, which disagrees with our previous findings in the lit mice, and highlights potentially important differences between the two mouse models. Nonetheless, once-daily CV1574-1 fusion protein treatment corrected much of the growth plate height deficit without increasing cell proliferation in a non-targeted tissue in this pegvisomant model, providing further supporting evidence that this cartilage-targeted IGF-1 fusion protein is efficacious in both GH-deficiency as well as GH-insensitivity. Our findings suggest that a cartilage-targeted IGF-1 fusion proteins may show greater efficacy than IGF-1 itself in children with GH-insensitivity syndrome without increasing off-target effects. The findings further suggest that cartilage-targeted IGF-1 may require less frequent administration than is needed for IGF-1 itself.

It has been reported that, during long-term treatment with IGF-1, approximately half of treated patients develop hypoglycemia (13). We therefore compared the induction of hypoglycemia after injection of CV1574-1 or IGF-1 by monitoring blood glucose levels in fasted mice. Unlike IGF-1, which lowered blood glucose levels shortly after subcutaneous injection, CV1574-1 injection did not induce hypoglycemia. Our findings therefore suggest that CV1574-1 fusion protein has a lower risk of acute adverse hypoglycemic effects compared to commercially available IGF-1.

Lastly, we explored the molecular mechanisms by which cartilage-targeting improves the efficacy of CV1574-1 fusion protein. We found that transient exposure of RCS cells to CV1574-1 induced more prolonged phosphorylation of AKT compared to IGF-1 alone. The antibody fragment in CV1574-1 specifically targets matrilin-3 within cartilage matrix. To prevent this targeting, we knocked out matrilin-3 in RCS cells and found that absence of matrilin-3 abolished the prolonged AKT simulation by CV1574-1 compared to IGF-1. These findings suggest that matrilin-3 targeting allows CV1574-1 to stay in proximity to the cell surface for a longer period of time, extending the duration of IGF-1 receptor activation. Whether the fusion protein is able to bind matrilin-3 and the IGF-1 receptor simultaneously or whether the fusion protein is released from matrilin-3 before binding the receptor remains to be determined.

In conclusion, our study provides further evidence that cartilage-targeted therapy has the potential to provide a new pharmacological approach for the treatment of childhood linear growth disorders, including Laron syndrome, skeletal dysplasias, and growth failure due to systemic disease.

## Data Availability

The raw data supporting the conclusions of this article will be made available by the authors, without undue reservation.
