# Supplementary material for: Efficacy of cartilage-targeted IGF-1 in a mouse model of growth hormone insensitivity
Source: Front Endocrinol (Lausanne). 2025 Jan 9;15:1523931. doi: 10.3389/fendo.2024.1523931 (PMC11756323; doi:10.3389/fendo.2024.1523931)
Supplement: Supplementary file 1 [file DataSheet1.docx]

Supplementary Material

**Title:** Efficacy of cartilage-targeted IGF-1 in a mouse model of growth hormone insensitivity

**Authors:** Krishma Tailor^1,2^, Janine van Ree^2^, Timothy Stowe^2^, Brit Ventura^2^, Connor Sisk^1^, Joanna Courtis^1^, Anna Camp^1^, Fatima Elzamzami^1^, Jan van Deursen^2^, Robert O'Brien^2^, Jeffrey Baron^1^, Julian C. Lui^1^

## Supplementary Figures


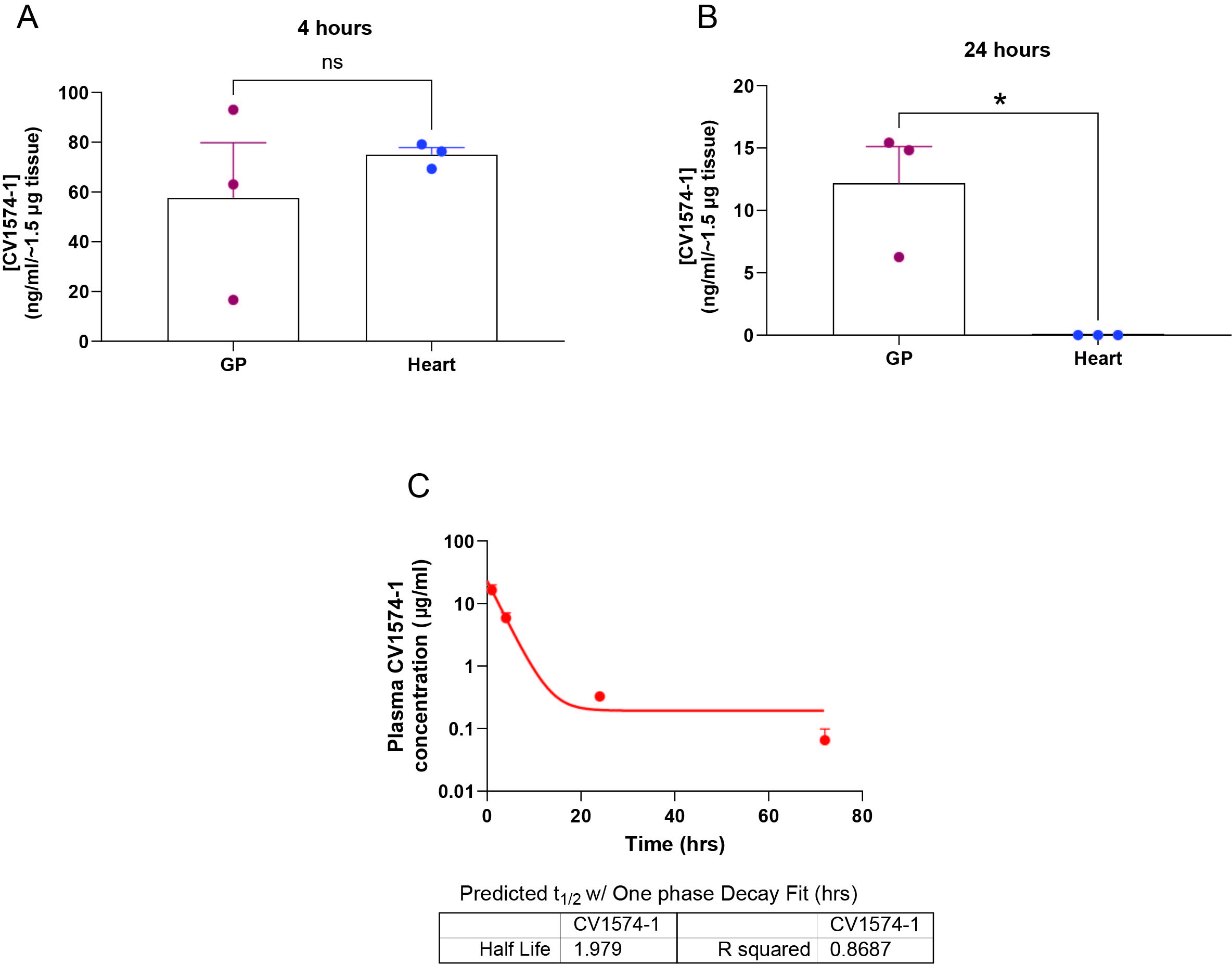


**Supplementary Figure 1.** **Pharmacokinetic characterization of CV1574-1.** The accumulation of CV1574-1 in the targeted tissue, growth plate (GP), and a non-targeted tissue, heart, at (A) 4 hours and (B) 24 hours, was measured using a hIgG ELISA. Mean± SEM, * P<0.05, by unpaired t-test. N=3 pups/group. (C) Systemic pharmacokinetics of CV1574-1 in plasma. Plasma CV1574-1 was measured by ELISA, and a one-phase decay fit test was performed using non-linear regression analysis in GraphPad Prism. The plasma concentration of the CV1574-1 decreased over time following a one-phase exponential decay pattern (R² = 0.8687). The calculated decay rate constant (K) was 0.3502, with a half-life of CV1574-1 of approximately 2 hours.


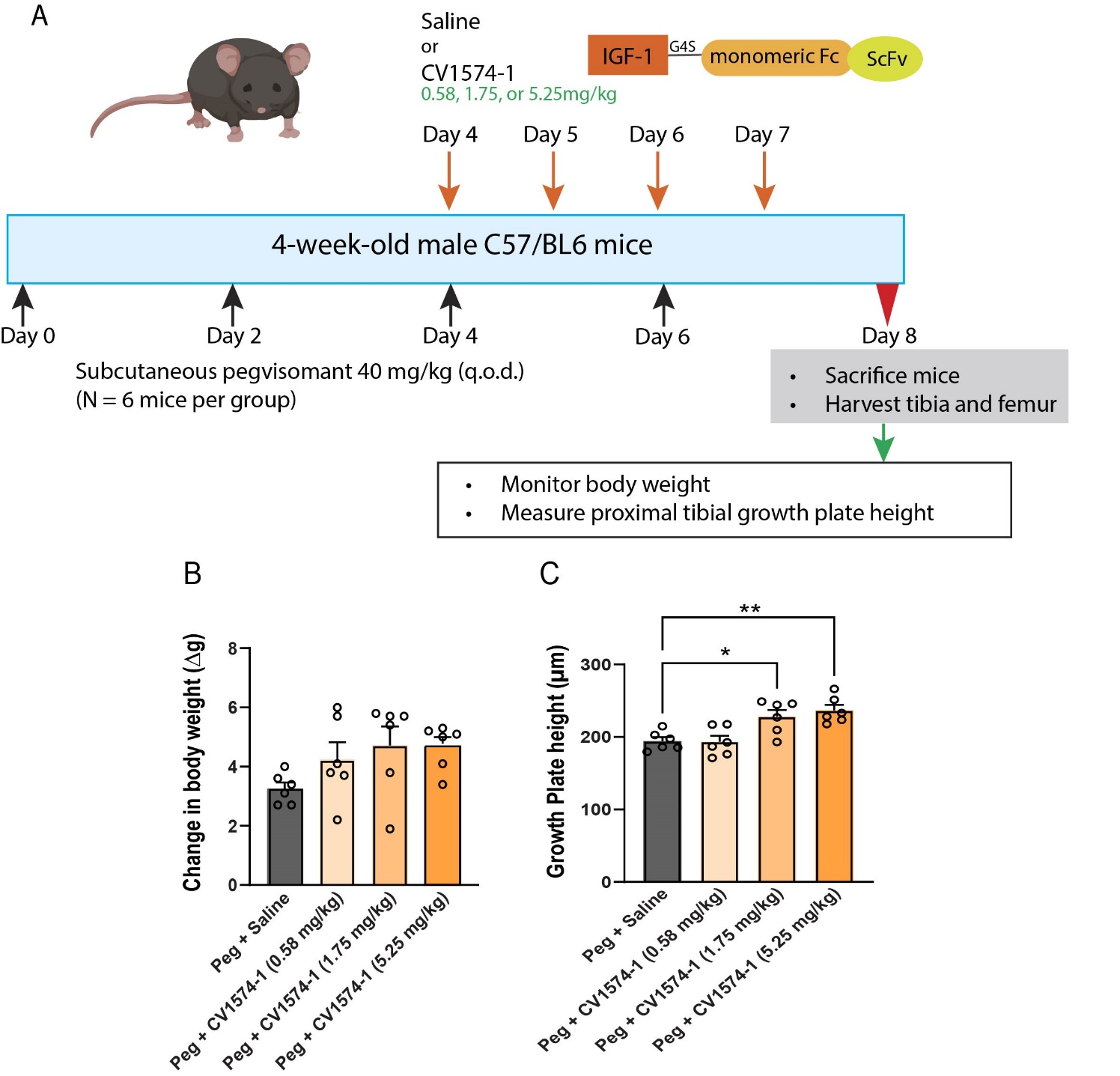


**Supplementary Figure 2.** **Effect of CV1574-1 in pegvisomant mouse model.** (A) 5-week-old pegvisomant-treated male mice received daily subcutaneous CV1574-1 at the indicated doses. Body weight (B), and growth plate height of the proximal tibia (C) are shown as mean± SEM, * P<0.05, **P<0.01 by one-way ANOVA followed by multiple comparisons between all groups. P-value corrected by Dunnett’s test. N=6 mice per group.


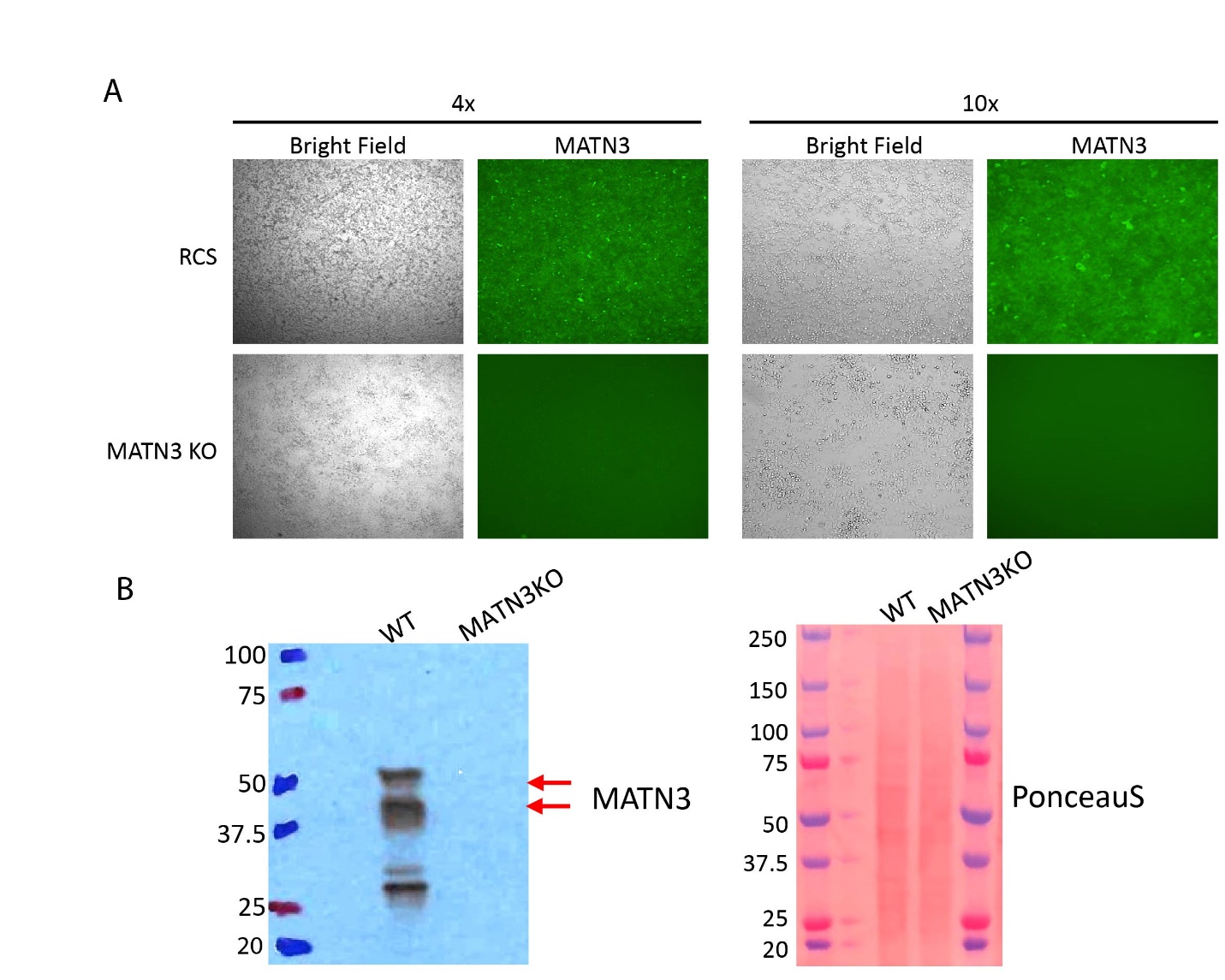


**Supplementary Figure 3.** **Confirmation of matrilin-3 knockout in RCS cells.** The absence of endogenous matrilin-3 in RCS cells after CRISPR-Cas9 ablation was confirmed by the absence of positive staining with anti-matrilin-3 antibody, observed by immunofluorescence staining (A) and by western blot (B).
